# Suicidal ideation among Chinese methadone-maintained patients: prevalence and correlates

**DOI:** 10.18632/oncotarget.21032

**Published:** 2017-09-16

**Authors:** Yan-Min Xu, Bao-Liang Zhong, Wen-Cai Chen, Jun-Hong Zhu, Jin Lu

**Affiliations:** ^1^ Affiliated Wuhan Mental Health Center (The Ninth Clinical School), Tongji Medical College of Huazhong University of Science & Technology, Wuhan, Hubei Province, China; ^2^ Department of Psychiatry, The First Affiliated Hospital of Kunming Medical University, Kunming, Yunnan Province, China

**Keywords:** suicidal ideation, methadone maintenance treatment, prevalence, correlate

## Abstract

Heroin users are at high risk for suicide. However, the epidemiological profile of suicidal behaviors in Chinese methadone-maintained patients remains largely unknown. This study determined the prevalence and correlates of suicidal ideation among Chinese methadone-maintained patients. A total of 603 methadone-maintained patients were consecutively recruited from three methadone maintenance treatment (MMT) clinics in Wuhan, China, and administered with standardized questionnaires to collect sociodemographic, clinical, and psychological data. Suicidal ideation was measured with a single self-report question “Have you ever thought about committing suicide?”. Depression and anxiety were assessed with Zung’s Self-rating Depression Scale and Zung’s Self-rating Anxiety Scale, respectively. The one-month and lifetime prevalence rates of suicidal ideation were 17.9% and 58.9%, respectively. In multiple logistic regression, lifetime suicidal ideation was significantly associated with female (OR: 1.69), an educational attainment of primary school and below (OR: 1.47), fair and poor interpersonal relationship (OR: 2.20), a history of injecting heroin (OR: 1.60), depression (OR: 1.38), and anxiety (OR: 4.00). Methadone-maintained patients of MMT clinics have a high prevalence of suicidal ideation and therefore at high risk for suicide. Suicide prevention efforts at MMT clinics should include periodic evaluation of suicidality, psychosocial supports, and, when necessary, psychiatric treatment and crisis intervention.

## INTRODUCTION

Methadone maintenance treatment (MMT) is widely recognized as an effective treatment for reducing opioid addiction and its negative health and social consequences such as the HIV/AIDS epidemic and crimes [1]. China, with the largest population of drug users globally, initiated its MMT program in 2004, and, until now, its MMT has become the world’s largest MMT program [2, 3]. According to official statistics, by 2014, there had been 765 community-based MMT clinics providing services to 192920 illicit drug (mainly heroin) users in 28 provinces of the mainland China [4]. Nevertheless, MMT in China faces many challenges, such as the underrecognition and undertreatment of psychosocial problems in methadone-maintained patients [5, 6].

Opiate addicts are at high risk for suicide, and the risk is particularly high among those with comorbid depression [7]. For example, patients with heroin use disorders are 14 times more likely than members of the general population to commit suicide, and heroin users with major depression is 5.5 times more likely than those without major depression to attempt suicide in the preceding year [7–9]. Because there is evidence that MMT effectively improves the social well-being and mental and physical quality of life of heroin addicts [1, 10], the risk for suicide in individuals who are admitted to MMT may have declined. However, a cross-sectional comparison study still reported comparable one-year and one-month prevalence rates of suicide attempts between heroin-dependent patients receiving methadone/buprenorphine maintenance treatment and those not receiving any treatment [8]. Findings from an 11-year follow-up study further show that there was no reduction in the prevalence of lifetime attempted suicide in heroin users of drug treatment facilities (34% at baseline and 42.2% at the end of follow-up) [8, 11], suggesting that the risk for suicide is persistently high among individuals receiving treatment for heroin dependence. This phenomenon could be explained by the very high prevalence of psychosocial risk factors for suicide and inadequate treatment of psychosocial problems in this patient population: for example, 33.8-68% MMT patients have clinically significant depressive symptoms [12–15] and many depressed heroin users receive no treatment [12, 16].

In contrast to the numerous studies on suicidal behaviors in the general population, suicidal behaviors in patients with opioid dependence has received less research attention, in particular for Chinese heroin addicts [7, 17]. In China, there have been a limited number of studies investigating suicidal behaviors of heroin addicts [15, 18–23]. Due to heterogeneity in methodology (i.e., convenient vs. consecutive sampling) and measures of suicidal behaviors (i.e., “suicidal thoughts” item of the Symptom Checklist-90 vs. a self-designed question), these studies reported a wide range of prevalence rates of lifetime suicidal ideation (from 13.9% to 66.7%) [21] and attempted suicide (from 0.66% to 16.5%) [18, 23]. Because most existing studies focused on suicidal behaviors of Chinese heroin users in compulsory detoxification settings [17], their findings can not be generalized to heroin users of Chinese MMT clinics. Moreover, only two previous studies had studied socio-demographic correlates of suicidal ideation among Chinese heroin users [15, 19], a detailed epidemiological profile such as psychosocial factors of suicidal behaviors remains largely unknown. In addition, although the major risk factors for suicidal behaviors of the general population also apply to heroin users, it is unclear that whether clinical factors (heroin use characteristics and treatment) add additional risk to their suicidal behaviors [9, 17].

Suicidal ideation refers to “thoughts of engaging in any suicide-related behavior, ranging from transient and intermittent thoughts about death and more severe rumination and creation of a plan to kill oneself” [24]. Evidence shows that suicidal ideation predicts subsequent attempted and completed suicide [25, 26]. Therefore, examining the prevalence and correlates of suicidal ideation in addiction treatment practice is important for the early recognition of patients at risk for suicide. This study was carried out to determine the epidemiology of suicidal ideation in Chinese methadone-maintained patients.

## RESULTS

In total, 652 eligible patients were invited to participant in the study, of whom 603 (92.5%) completed the study. The mean age of the 603 completers was 38.1 years (Standard Deviation [SD] = 7.0), and 69.8% were males. The majority of patients (84.1%) injected heroin before MMT, and the average dose and duration of methadone were 69.5 mg/d (SD=29.6) and 24.6 months (SD=11.0), respectively. Other detailed sociodemographic, clinical, and psychological characteristics are displayed in Table 1.

**Table 1:** Characteristics of participants and prevalence rates of suicidal ideation by variables

Characteristics		n	One-month suicidal ideation				Lifetime suicidal ideation			
			Number	%	χ2	P	Number	%	χ2	P
Gender	Male	412	71	17.2	0.083	0.773	230	55.8	4.891	0.027
	Female	191	34	17.8			125	65.4		
Age (years)	20-39	327	56	17.1	0.011	0.919	196	59.9	0.363	0.547
	40-50	276	49	17.8			159	57.6		
Education	Primary school and below	64	17	26.6	2.947	0.086	50	26.6	11.866	<0.001
	Middle school and above	539	88	16.3			305	16.3		
Marital status	Married	295	42	14.2	3.196	0.074	168	56.9	0.882	0.348
	Non-married*	308	63	20.5			187	60.7		
Employment	Yes	317	38	12.0	10.889	0.001	177	55.8	1.887	0.171
	No	286	67	23.4			178	62.2		
Self-rated economic status	Good	149	14	9.4	17.838	<0.001	79	53.0	16.746	<0.001
	Fair	314	49	15.6			171	54.5		
	Poor	140	42	30.0			105	75.0		
Interpersonal relationship	Good	170	14	8.2	14.071	<0.001	71	41.8	22.415	<0.001
	Fair and poor^#^	433	91	21			284	65.6		
Route of past heroin administration	Smoking	96	7	7.3	8.246	0.004	37	38.5	16.652	<0.001
	Injecting	507	98	19.3			318	62.7		
Duration of past heroin use (years)	≤10	373	70	18.8	1.058	0.304	224	60.1	0.495	0.482
	>10	230	35	15.2			131	57.0		
Duration of MMT (months)	≤24	240	47	19.6	1.561	0.212	135	56.3	0.949	0.331
	>24	363	58	16.0			220	60.6		
Methadone dose (md/d)	≤70	273	37	13.6	4.909	0.027	141	51.6	9.484	0.002
	>70	330	68	20.6			214	64.8		
Depressive symptoms	No	399	55	13.8	7.844	0.005	213	53.4	11.861	0.001
	Yes	204	50	24.5			142	69.6		
Anxiety symptom	No	384	28	7.3	68.693	<0.001	190	49.5	33.932	<0.001
	Yes	219	77	35.1			165	75.3		

*“Non-married” includes never-married, remarried, cohabitating, separated/divorced, and widowed.

^#^ Because very few patients rated their interpersonal relationship as “poor”, “fair” and “poor” were merged into one category.

In total, 105 and 355 patients endorsed one-month and lifetime suicidal ideation, respectively. The prevalence rates of one-month and lifetime suicidal ideation were 17.9% and 58.9%, respectively. Results of Chi-square test (Table 1) showed that, patients with one-month suicidal ideation were more likely to be unemployed, rate their economic status as “poor” or “fair”, have fair and poor interpersonal relationship, inject heroin before being admitted to MMT, take a high dose of methadone, be depressed, and be anxious, while patients with lifetime suicidal ideation were more likely to be females, have educational attainment of primary school and below, have “poor” or “fair” economic status, rate their interpersonal relationship as “fair” and “poor”, have a history of injecting heroin, take a high dose of methadone, and have more depressive and anxiety symptoms.

Multiple logistic regression (Table 2) revealed that, one-month suicidal ideation was significantly associated with poor economic status (odds ratio [OR]: 3.22), fair and poor interpersonal relationship (OR: 1.39), a history of injecting heroin (OR: 1.39), depression (OR: 1.50), and anxiety (OR: 6.43), while lifetime suicidal ideation was significantly associated with female (OR: 1.69), an educational attainment of primary school and below (OR: 1.47), fair and poor interpersonal relationship (OR: 2.20), a history of injecting heroin (OR: 1.60), depression (OR: 1.38), and anxiety (OR: 4.00).

**Table 2:** Multivariate binary logistic regression on factors significantly associated with suicidal ideation

Variables		One-month suicidal ideation					Lifetime suicidal ideation				
		Coefficient	Standard error	Wald χ2	P	OR(95%CI)	Coefficient	Standard error	Wald χ2	P	OR(95%CI)
Gender	Male	---	---	---	---	---	1				
	Female	---	---	---	---	---	0.524	0.21	6.252	0.012	1.69(1.12,2.55)
Education	Middle school and above	---	---	---	---	---	1				
	Primary school and below	---	---	---	---	---	0.962	0.369	6.803	0.009	1.47(1.20,2.20)
Self-rated economic status	Good	1					---	---	---	---	---
	Poor	1.168	0.426	7.516	0.006	3.22(1.40,7.41)	---	---	---	---	---
Interpersonal relationship	Good	1					1				
	Fair and poor^#^	1.141	0.385	8.716	0.003	3.13(1.47,6.66)	0.790	0.217	13.262	<0.001	2.20(1.44,3.37)
Route of past heroin administration	Smoking	1					1				
	Injecting	1.106	0.465	5.657	0.017	1.39(1.14,2.28)	0.761	0.256	8.819	0.003	1.60(1.33,2.16)
Depressive symptoms	No	1					1				
	Yes	0.899	0.425	4.468	0.035	1.50(1.19,2.55)	1.128	0.387	8.501	0.004	1.38(1.16,2.00)
Anxiety symptoms	No	1					1				
	Yes	1.861	0.321	33.495	<0.001	6.43(3.42,12.06)	1.385	0.261	28.291	<0.001	4.00(2.40,6.65)

^#^Because very few patients rated their interpersonal relationship as “poor”, “fair” and “poor” were merged into one category.

## DISCUSSION

Because lifetime prevalence evaluates long-term healthcare needs and one-month prevalence reflects current healthcare needs for suicide prevention, a dual timeframe for suicidal ideation was used for this study. Our study is one of the very few studies that investigated the epidemiology of suicidal ideation among Chinese heroin-dependent patients receiving MMT. We found that 17.9% and 58.9% of the Chinese methadone-maintained patients had thoughts about committing suicide during the past month and their lifetime, respectively. The lifetime prevalence (58.9%), even the one-month prevalence (17.9%), of suicidal ideation in this patient population is much higher than the lifetime prevalence in Chinese general population, which is estimated to be 3.9% [27]. Compared to studies conducted among heroin users, the prevalence of suicidal ideation in our sample of methadone-maintained patients is lower than that of heroin-dependent patients of compulsory detoxification settings (one-month: 17.4% vs. 33%; lifetime: 58.9% vs. 65%) [20], but higher than that of Australian heroin users (one-month: 17.4% vs. 10.4%) [11]. These discrepancies may be ascribed to differences in clinical settings and patients’ characteristics: for example, patients of Chinese compulsory detoxification centers may suffer more intense pain than those of MMT clinics due to acute withdrawal, and therefore a relatively low prevalence of suicidal ideation was observed in MMT patients.

In the Chinese general population, lifetime suicidal ideation is more prevalent in females than males, with a female-male ratio being 1.7 [27]. Similarly, we found that female gender was independently associated with 1.69-fold increase in risk of lifetime suicidal ideation in MMT patients. Consistent with the elevated risk of suicidal behaviors in individuals with low socio-economic status [28, 29], we found that a low level of education and poor economic status were independent predictors of suicidal ideation. These associations might be explained by low socioeconomic status’ negative effects on health and wellbeing, which, in turn, place individuals at increased risk for suicidal behaviors.

Prior studies have reported that social isolation is a significant predictor of suicide and attempted suicide in heroin users [9, 25] and social isolation is significantly associated with suicidal ideation of adolescents [30]. Similar to these earlier studies, we found a significant relationship between poor interpersonal relationship and suicidal ideation in methadone-maintained patients: individuals who poorly get along with others are at risk for social isolation and loneliness due to their small social networks and inadequate social support [31].

Because patients who injected heroin before MMT have more physical conditions such as HIV/AIDS or Hepatitis [32], the significant link between injecting heroin and suicidal ideation might be an indirect reflection of the association between these major medical conditions and suicidal behaviors.

Finally, we found that depression and anxiety were two significant contributing factors to suicidal ideation in MMT patients, with anxiety being the strongest among all contributing factors. This finding is a little different from what is generally acknowledged, that is, depression is the strongest predictor of suicidality in the general population [33, 34]. Because post-traumatic stress disorder (PTSD) is common among Chinese heroin-dependent patients (8.1% prevalence) [35] and individuals with PTSD are at greater risk for suicidal thoughts and behaviors [36], the strong association between anxiety and suicidal ideation might be a reflection the influence of PTSD on suicidal behaviors.

This study has several limitations. First, our heroin user sample was recruited from MMT clinics of a large city in south central China, heroin users of other institutions, i.e., compulsory detoxification centers and psychiatric hospitals, and other cities were not included, potentially limiting the generalizability of our findings. Second, owing to the observational nature of this study, the causal relationships between suicidal ideation and identified associated factors need to be further examined in longitudinal studies. Third, some major medical conditions such as HIV and HCV are also associated with an elevated risk of suicidal behaviors, but we did not collect data on the physical health status of these patients.

In summary, suicidal ideation are extremely prevalent among Chinese methadone-maintained patients, indicating the high healthcare needs for suicide prevention in Chinese MMT clinics. There is an urgent need for health authorities and health workers to address the epidemic of suicidal behaviors in methadone-maintained patients. Amongst methadone-maintained patients, a range of factors, in particular modifiable psychosocial factors, are associated with suicidal ideation. Efforts to prevent suicide in MMT clinics may be useful to target on those who are women and have a low socioeconomic status (i.e., a low level of education and financial difficulties), poor interpersonal relationship, a history of injecting heroin, and clinically significant depressive and anxiety symptoms. Services for methadone-maintained patients should include periodic risk assessment for suicide, expanded social supports that specifically focus on improving interpersonal relationship, and, when necessary, psychiatric assessment and treatment and crisis intervention.

## MATERIALS AND METHODS

### Subjects

The data presented in this study were collected between 2009 and 2010 as part of the project “Mental health promotion for patients of MMT clinics in Wuhan, China: a comprehensive study” [37]. This project is a cross-sectional survey of heroin-dependent patients, consecutively recruited from three MMT clinics in Wuhan, China. Eligibility criteria for subjects were 1) aged over 19 years, 2) met DSM-IV criteria for a lifetime diagnosis of heroin addiction, 3) receiving MMT, 4) able to complete the self-completed questionnaire, and 5) agreed to take part in the study. Details of subjects recruitment, measures, and quality control have been published elsewhere [12].

Before the beginning of the survey, ethics approvals were obtained from the Ethics Committee of Wuhan Mental Health Center. All participants provided written informed consent.

### Procedures and assessments

Socio-demographic variables collected in the questionnaire included gender, age, education, marital status, employment status, financial status, and interpersonal relationship. We assessed interpersonal relationship with a single question (“Overall, how did you get along with others, including family and non-family associates?”) on a 3-point scale: 1=poor, 2=fair, 3=well [38].

Clinical factors were main route of past heroin administration, duration of past heroin use, duration of MMT, and methadone dose (mg/d).

Psychological factors included depressive and anxiety symptoms, which were assessed with Zung’s Self-rating Depression Scale (SDS) and Zung’s Self-rating Anxiety Scale (SAS) [39], respectively. The total scores of both scales range from 20 to 80, with higher scores indicating more depressive or anxiety symptoms. A cut-off scores of 40 in Chinese SDS and 43 in Chinese SAS are used to determine clinically significant depressive and anxiety symptoms [39], respectively.

One question about suicidal ideation was adapted from the National Comorbidity Survey [40]: “Have you ever thought about committing suicide?” If the respondent endorsed having ever thought about suicide, he/she would be recorded as having lifetime suicidal ideation, and the investigator would further ask: “When was the last time?”. If a response was affirmative for any time during the prior month, the respondent was additionally recorded as having one-month suicidal ideation.

Trained investigators were assigned to assist illiterate respondents to complete the questionnaire. Before the survey, a pilot study was conducted among a sample 48 MMT patients to test the feasibility of our study procedures. The survey questionnaire was also finalized after the pilot study.

### Statistical analysis

We calculated the prevalence rates of one-month and lifetime suicidal ideation in different cohorts according to subjects’ socio-demographic, clinical, and psychological characteristics. Chi-square test was used to compare prevalence rates between different cohorts. Multivariable logistic regression model with a backward stepwise entry of significant variables in the Chi-square test was used to identify factors significantly associated with one-month and lifetime suicidal ideation, respectively. ORs and 95% confidence intervals (CIs) were used to quantify the associations between factors and suicidal ideation. The statistical significance level was set at p<0.05 (two-sided). SPSS software version 19.0 package was used for analyses.
